# Nexilin/*NEXN* controls actin polymerization in smooth muscle and is regulated by myocardin family coactivators and YAP

**DOI:** 10.1038/s41598-018-31328-2

**Published:** 2018-08-29

**Authors:** Baoyi Zhu, Catarina Rippe, Johan Holmberg, Shaohua Zeng, Ljubica Perisic, Sebastian Albinsson, Ulf Hedin, Bengt Uvelius, Karl Swärd

**Affiliations:** 1 Department of Experimental Medical Science, Lund University, SE-221 84 Lund, Sweden; 2 Department of Urology, the Sixth Affiliated Hospital of Guangzhou Medical University (Qingyuan People’s Hospital), 511518 Guangdong, China; 3 Department of Molecular Medicine and Surgery, Karolinska Institutet, Stockholm, Sweden; 4 Department of Clinical Science, Section of Urology, Lund University, SE-221 84 Lund, Sweden

## Abstract

Nexilin, encoded by the *NEXN* gene, is expressed in striated muscle and localizes to Z-discs, influencing mechanical stability. We examined Nexilin/*NEXN* in smooth muscle cells (SMCs), and addressed if Nexilin localizes to dense bodies and dense bands and whether it is regulated by actin-controlled coactivators from the MRTF (*MYOCD*, *MKL1*, *MKL2*) and YAP/TAZ (*YAP1* and *WWTR1*) families. *NEXN* expression in SMCs was comparable to that in striated muscles. Immunofluorescence and immunoelectron microscopy suggested that Nexilin localizes to dense bodies and dense bands. Correlations at the mRNA level suggested that *NEXN* expression might be controlled by actin polymerization. Depolymerization of actin using Latrunculin B repressed the *NEXN* mRNA and protein in bladder and coronary artery SMCs. Overexpression and knockdown supported involvement of both YAP/TAZ and MRTFs in the transcriptional control of *NEXN*. YAP/TAZ and MRTFs appeared equally important in bladder SMCs, whereas MRTFs dominated in vascular SMCs. Expression of *NEXN* was moreover reduced in situations of SMC phenotypic modulation *in vivo*. The proximal promoter of *NEXN* conferred control by MRTF-A/*MKL1* and *MYOCD*. *NEXN* silencing reduced actin polymerization and cell migration, as well as SMC marker expression. *NEXN* targeting by actin-controlled coactivators thus amplifies SMC differentiation through the actin cytoskeleton, probably via dense bodies and dense bands.

## Introduction

Phenotypic modulation is a process in which smooth muscle cells (SMCs) toggle between two or more distinct phenotypes. The two classical phenotype opposites are characterized by contractility and quiescence on the one hand, and proliferation and matrix synthesis on the other hand. Under certain conditions, such as during embryonic development and adaptation to mechanical forces, growth and contractile differentiation can occur simultaneously. Switching between phenotypes is a tightly orchestrated, but reversible, process seen in atherosclerosis and neointima formation^1,2^, gastrointestinal^3^ and urinary bladder^4^ pathology, and which depends on myocardin related transcription factors (MRTFs), in particular myocardin (*MYOCD*)^1,2,5–9^. MRTFs (*MYOCD*, MRTF-A/*MKL1*and MRTF-B/*MKL2*) act through the serum response factor (*SRF*) and so called CArG boxes^10^ in the promoters of target genes^5,6^. In certain contexts MRTFs may form other transcriptional complexes, including those containing YAP/TAZ/TEAD^11–13^. An important physiological control mechanism for MRTFs and YAP/TAZ is the ratio of filamentous (F-) to globular (G-) actin^5,13,14^. For MRTF-A and MRTF-B, the mechanism involves G-actin-dependent cytoplasmic sequestration and reduced nuclear entry. Gene targets of MRTFs include ion channels^15–17^, kinases that activate contraction^18^, and myofilament constituents such as myosin, actin and calponin^19,20^. Target genes of MRTFs are thus involved in numerous aspects of the SMC activation process. MRTF/SRF signaling also plays roles for differentiation and function of striated muscle^21–24^, and cardiomyocyte-specific deletion of myocardin, for example, leads to dilated cardiomyopathy and heart failure^25^.

Despite the central importance of MRTFs in both smooth and striated muscle, there are clear morphological differences between these muscle types. One difference relates to the organization of the contractile apparatus, which in striated myofibrils is built on sarcomeres stretching between Z-discs (or lines) in a periodic fashion. Z-discs are complex but morphologically well-defined structures^26^ that anchor actin filaments of adjacent sarcomeres. Z-discs are absent in SMCs, but functional equivalents are dense bodies, which are scattered throughout the cytoplasm. Such electron dense structures also occupy space at the SMC plasma membrane, and are referred to as surface dense bands^27^. It has been shown that actin filaments at dense bodies point outward, similar to how actin is organized at the Z-disc^28^. Dense bodies moreover contain α-actinin^29^, similar to Z-discs^30^. In addition to α-actinin and actin, dense bands in SMCs contain vinculin, which anchors the cells via integrins to the extracellular matrix. Vinculin-containing dense bands and non-overlapping caveolae-domains create a bipartite organization of the plasma membrane^31^ that is lost when SMCs are isolated from tissue^32^.

Nexilin, encoded by the *NEXN* gene, is an actin filament-binding protein represented by two splice variants, localizing to cell-matrix junctions in cultured cells^33^. In cardiomyocytes Nexilin is localized at Z-discs and helps maintain Z-disc stability^34^. Human *NEXN* mutations, and *NEXN* gene disruption in mice, cause Z-disc destabilization and dilated^34,35^ or hypertrophic^36^ cardiomyopathy. Nexilin localization and function in differentiated SMCs *in situ* has not been studied. Early work indicated that *NEXN* expression in cultured SMCs is reduced and increased, respectively, by platelet-derived growth factor and Gα16^37^. More recently, single nucleotide polymorphisms near *NEXN* were found to associate with coronary artery disease^38^, and manipulation of *NEXN* influenced expression of myocardin and SRF along with SMC differentiation markers^38,39^, but the underlying mechanism was not characterized.

In the present study we have addressed the hypothesis that Nexilin associates with dense bodies and dense bands in SMCs. We also aimed to test if *NEXN* is regulated by actin polymerization and actin-sensitive transcriptional coactivators via its proximal promoter, and if it behaves as an SMC marker during phenotypic modulation *in vivo*. Our work demonstrates that Nexilin is highly expressed in SMCs in the cardiovascular system, in the gastrointestinal tract, and in the urinary bladder. Imaging suggests that Nexilin localizes to dense bands and dense bodies, and we find that its expression is controlled by MRTFs and YAP. We also demonstrate that Nexilin/*NEXN* silencing destabilizes F-actin, presumably by disrupting actin filament anchoring at dense bodies, and reduces SMC differentiation. Taken together, these findings argue that Nexilin is central for SMC structure and function.

## Results

### Nexilin/*NEXN* is highly expressed in SMCs and localizes to dense bands and dense bodies

**Figure 1:**
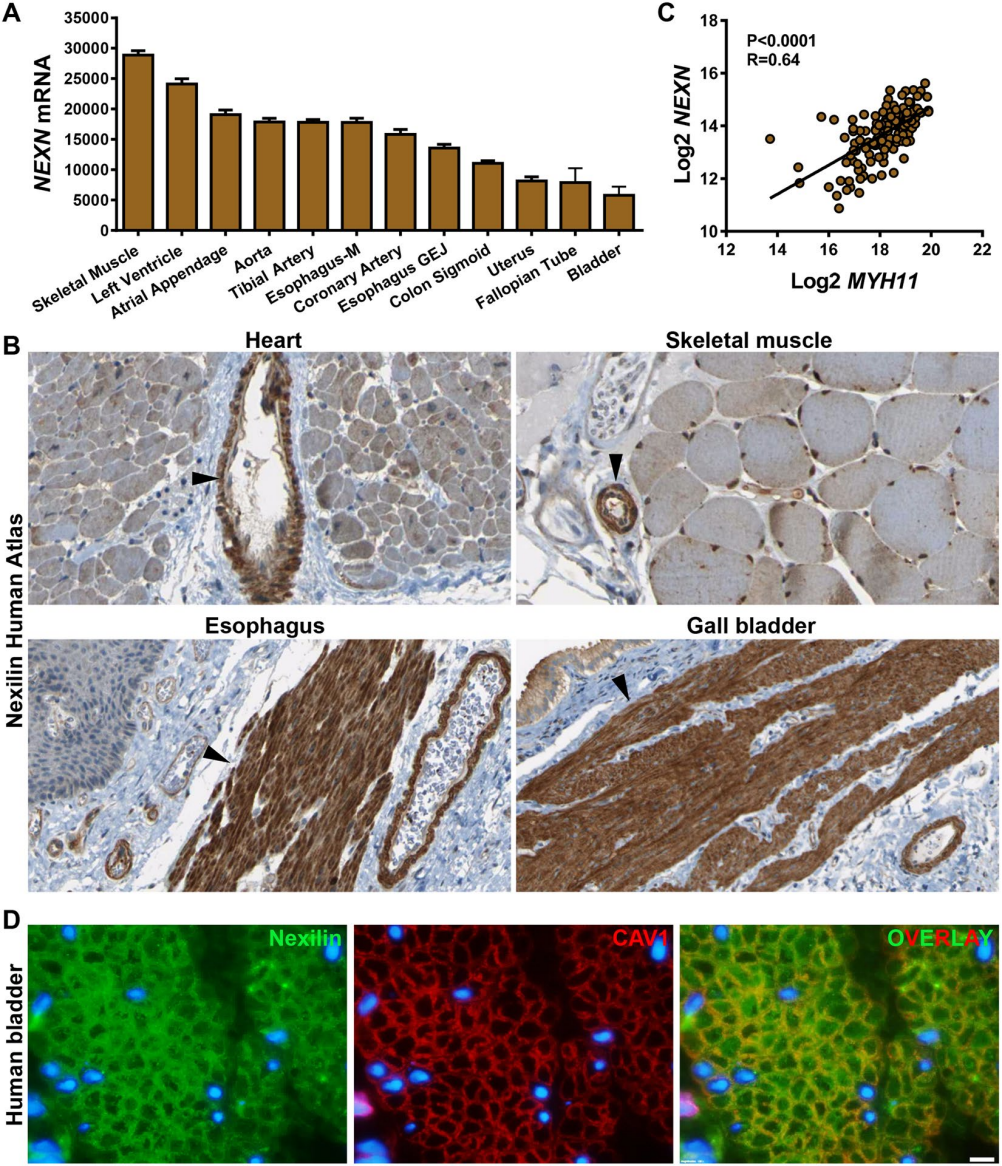
Nexilin/*NEXN* is expressed in striated and smooth muscle cells (SMCs) and correlates at the mRNA level with the smooth muscle myosin. Panel A shows RNA-Seq data (GTExPortal.org) for the top-twelve *NEXN*-expressing human tissues (N = 6–430, Esophagus-M: muscular layer, -GEJ: Gastroesophageal Junction). Panel B shows immunohistochemical staining for *NEXN* in human heart (top left), skeletal muscle (top right), esophagus (bottom left) and gall bladder (bottom right), all from the Human Protein Atlas (proteinatlas.org). Panel C shows correlation between smooth muscle myosin (*MYH11*) and *NEXN* in human coronary artery (N = 133, data from the GTExPortal). Panel D shows our own double staining for Nexilin (green, ab213628) and CAV1 (red) in cross-sectioned human urinary bladder SMCs (white scale bar represents 10 µm, N = 2) imaged in a conventional fluorescence microscope.

Our interest in Nexilin (*NEXN*) originated with a search for muscle-enriched genes using RNA (www.gtexportal.org)^40^ and protein (www.proteinatlas.org)^41^ expression databases. *NEXN* mRNA expression was high in skeletal muscle and heart, as expected, followed closely by SMC-containing tissues such as artery and esophagus (Fig. 1A shows the top-twelve *NEXN* expressing tissues). SMC-rich organs with a high volume fraction of mucosa, such as bladder and fallopian tube, had lower overall *NEXN* levels (Fig. 1A), and *NEXN* expression was nearly undetectable in the central nervous system and in blood (not depicted). Inspection of immunohistochemical staining for Nexilin in human heart and skeletal muscle (Fig. 1B, top panels, from the Human Protein Atlas) revealed staining in cardiomyocytes and skeletal muscle cells, but in both tissues, SMCs in large and small feeding arterioles also stained intensely (arrowheads, Fig. 1B). In esophagus and gall bladder SMCs, Nexilin staining was similarly intense (Fig. 1B, bottom panels), arguing for SMC-enrichment at the protein level. We next tested if *NEXN* correlates with the archetypal SMC marker *MYH11* at the mRNA level in arteries. Highly significant correlations were seen as illustrated for human coronary artery (Fig. 1C). Immunohistochemistry is poorly suited for subcellular resolution. We therefore also stained the human detrusor for Nexilin (Fig. 1D, green). The monoclonal antibody used (ab213628) stained puncta at the membrane and in the cytoplasm of SMCs. In some cells, nucleoli were also positive. Double staining for Nexilin and caveolin-1 (CAV1) (Fig. 1D, red) suggested some overlap (Fig. 1D, yellow in overlay) at the membrane, and none intracellularly. Specificity of the Nexilin antibody used in these experiments (ab213628) was supported by knockdown experiments (see below).

**Figure 2:**
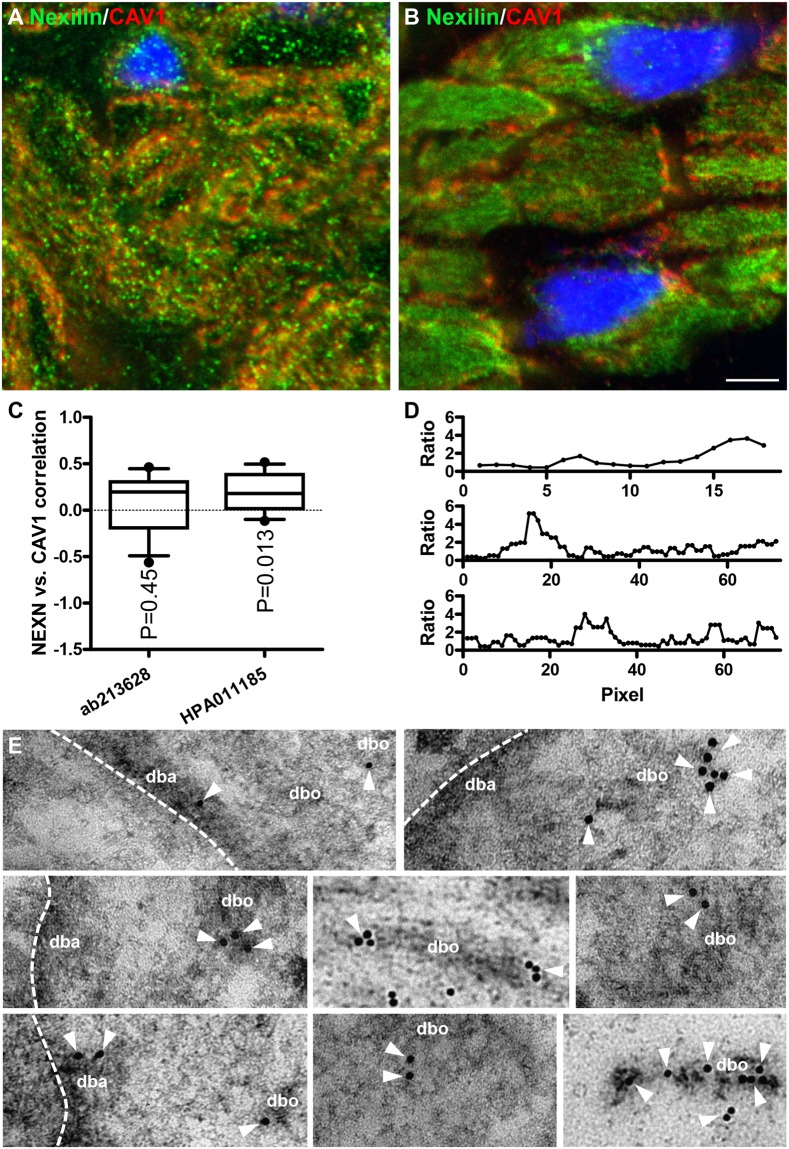
Nexilin localizes to puncta in the cytosol and at the membrane and is excluded from caveolae domains at the membrane. Nexilin (*NEXN*) and CAV1 immunofluorescence was imaged by confocal microscopy and using two different Nexilin antibodies (ab213628 in (**A)** and HPA011185 in (**B**). Both show punctate/granular distribution of Nexilin (green) in the cytoplasm and at the membrane in human bladder SMCs that overlaps poorly with CAV1 (red) staining. Correlation of the two labels (CAV1 and *NEXN*) indicated poor co-localization (panel C, R = 0.073 and 0.18, for the respective *NEXN* antibodies). The fluorescence intensity ratio for the two labels was also plotted for three membrane profiles (**D**). Panel E shows immuno-electron microscopy for Nexilin. The gold particle on the secondary antibody appears as a black sphere with a diameter of 10 nm, and examples of dense bodies (dbo) and dense bands (dba) are shown. White arrowheads highlight gold particles.

Low-resolution images can give false indications of colocalization. Thus, to further resolve Nexilin distribution in human detrusor SMCs, we used confocal imaging and compared *NEXN* distribution with that of caveolin-1 (CAV1). Two independent antibodies for Nexilin were used (ab213628 and HPA011185, Fig. 2A,B), and in both cases, no-primary and no-secondary controls were blank. Silencing experiments moreover supported specificity of both antibodies (see below and data not shown). The staining differed somewhat between the two antibodies, with the monoclonal antibody showing more punctate staining (Fig. 2A) and the polyclonal antibody showing more homogenous, albeit still focal, labelling (Fig. 2B). Both antibodies supported the presence of Nexilin in the cytoplasm and at the membrane. For one of them (HPA011185, B), longitudinal striations were occasionally seen in cells sectioned near the plasma membrane (top cell in B). Nexilin and Caveolin-1 (CAV1) showed poor co-localization in these high-resolution images (R = 0.073 ± 0.09, N = 11, for ab213628, and R = 0.181 ± 0.060, N = 11, for HPA011185) as indicated by correlation analysis of fluorescence intensities along profiles drawn to follow the membrane contour (Fig. 2C, one sample t-test versus 0 given). We also plotted the ratio of fluorescence intensities along the membrane and found that this ratio fluctuated considerably (Fig. 2D), as expected for proteins that occupy distinct membrane domains. Because the membrane of SMCs is separated into two domains, consisting of interdigitating caveolae domains and dense bands, this suggested that the membrane fraction of Nexilin may be present at dense bands. To directly address if Nexilin associates with dense bands and dense bodies, we performed immuno-electron microscopy. Solitary gold particles could occasionally be found in the cytoplasm, in nuclei and in mitochondria. In contrast, clusters of several particles were typically encountered at dense bodies and at dense bands (Fig. 2E shows eight examples). Indeed, we rarely observed clusters of two or more particles in structures other than dense bodies/dense bands.

### Nexilin/*NEXN* expression is sensitive to changes in actin polymerization

**Figure 3:**
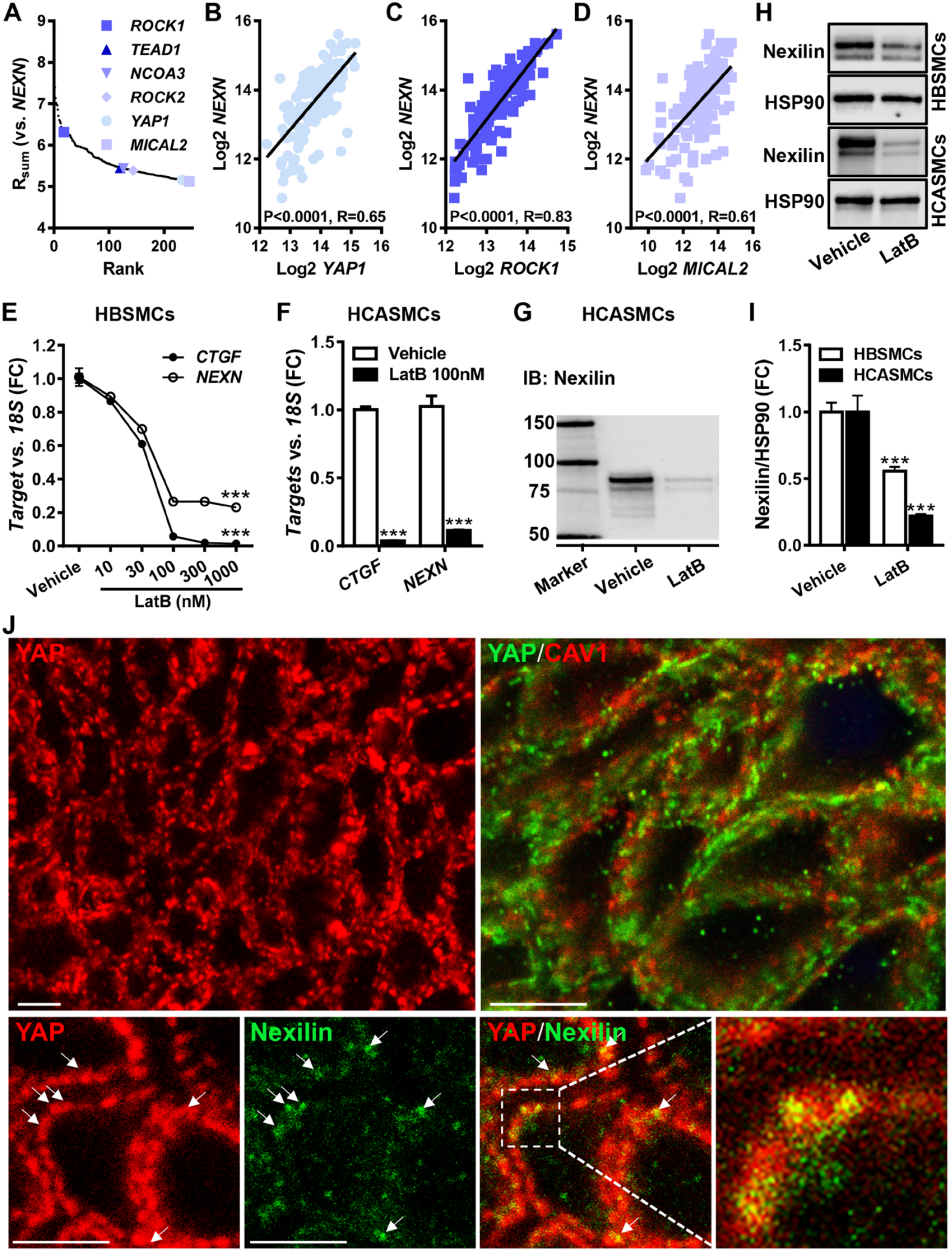
*NEXN* correlates with gene products that control and respond to changes in actin polymerization and Nexilin is reduced by depolymerization of actin. Correlations of *NEXN* versus all other RNAs in the top-ten *NEXN* expressing tissues were examined (data from GTExPortal). The sum of correlation coefficients for individual RNAs across tissues was calculated (R_sum_) and the positive extreme of this distribution was plotted (**A**). Actin controlling and responding gene products represented in the extreme are highlighted in blue colors. Examples of *NEXN* correlations in the human coronary artery (N = 133) are shown in panels B through (**D**). P-values and Spearman Rho values are given in the respective panels. Panels E and F show mRNA data for *NEXN* in cultured human bladder (HBSMCs, N = 8) and coronary artery (HCASMCs, N = 9) SMCs after treatment with Latrunculin B (LatB). Panels H and I show protein data for Nexilin/*NEXN* in the presence and absence of LatB (HBSMCs, 300 nM, N = 12; HCASMCs, 100 nM, N = 10). The top micrographs in panel J shows confocal imaging of YAP (red) on the left, and YAP (green) and CAV1 (red) on the right. The bottom row shows YAP (red) and Nexilin (green). The high magnification overlay at the bottom right shows partial colocalization of YAP and Nexilin at the cell membrane in yellow. All micrographs are from cross-sectioned HBSMCs and white scale bars represent 5 μm throughout.

We next reasoned that the high expression of *NEXN* in muscle might be governed by tissue-specific transcriptional control mechanisms. To approach the transcriptional regulation of *NEXN* we used an mRNA correlation approach that we have used previously^42,43^. Briefly, the *NEXN* mRNA was correlated with all other RNAs in the top-ten *NEXN* expressing tissues (GTEx data). The sum of correlation coefficients for individual tissue RNAs was then calculated, and the positive extreme (top 0.5%, Fig. 3A) of this distribution was inspected. *ROCK1* and *ROCK2*, both of which regulate actin polymerization in the cytosol were represented, as was *MICAL2*, which regulates actin polymerization in the nucleus. The actin-regulated coactivator *YAP1* and its binding partner *TEAD1* were similarly represented. Examples of correlations in the human coronary artery are depicted in Fig. 3B, C, and  D. MRTF-A/*MKL1* and myocardin/*MYOCD*, which are examined further below, were not present among the most tightly correlating RNAs (panel 3A, top 0.5%). This was due to lack of correlation in skeletal muscle, but both coactivators, and in particular *MYOCD*, correlated significantly with *NEXN* in arteries, in other SMC-rich tissues, and in the heart (*MYOCD* vs. *NEXN* in the coronary artery and left ventricle yielded Spearman Rho values of 0.55 and 0.48 and P < 0.0001 for both).

A possible explanation for the enrichment of gene products that control actin polymerization in the *NEXN* co-expression module is that *NEXN* gene activity is directly controlled by actin polymerization. To test this hypothesis, we treated human bladder (HBSMCs) and coronary artery SMCs (HCASMCs) with Latrunculin B (LatB), which depolymerizes actin. LatB reduced the *NEXN* mRNA level in both cell types (Fig. 3E,F). *CTGF*, an archetypal YAP/TAZ target, included as a positive control, was similarly reduced. We next examined the effect of LatB on the Nexilin protein level using western blotting. Nexilin migrated as two major bands between 75 and 100 kDa (Fig. 3G), consistent with previous work^33^, and both Nexilin bands were reduced in bladder and coronary artery SMCs treated with LatB (Fig. 3H,I).

In view of the potential role of YAP in the transcriptional control of *NEXN* we also stained cross-sectioned human bladder strips for YAP1 and examined its distribution using confocal microscopy. Strikingly, YAP had a heterogeneous membrane distribution in bladder SMCs (Fig. 3J, top left). YAP co-localized poorly with CAV1 (Fig. 3J, top right, R = 0.094 ± 0.042, N = 12). In contrast, double staining of YAP and Nexilin (Fig. 3J, bottom row), indicated co-localization at the membrane (R = 0.339 ± 0.051, N = 14). Indeed, co-localization at the membrane of YAP and Nexilin was significantly better than was co-localization of YAP and CAV1 (P = 0.0013). This suggests that YAP localizes at dense bands at the membrane similar to the membrane fraction of Nexilin.

### YAP and MRTFs stimulate Nexilin/*NEXN* expression

**Figure 4:**
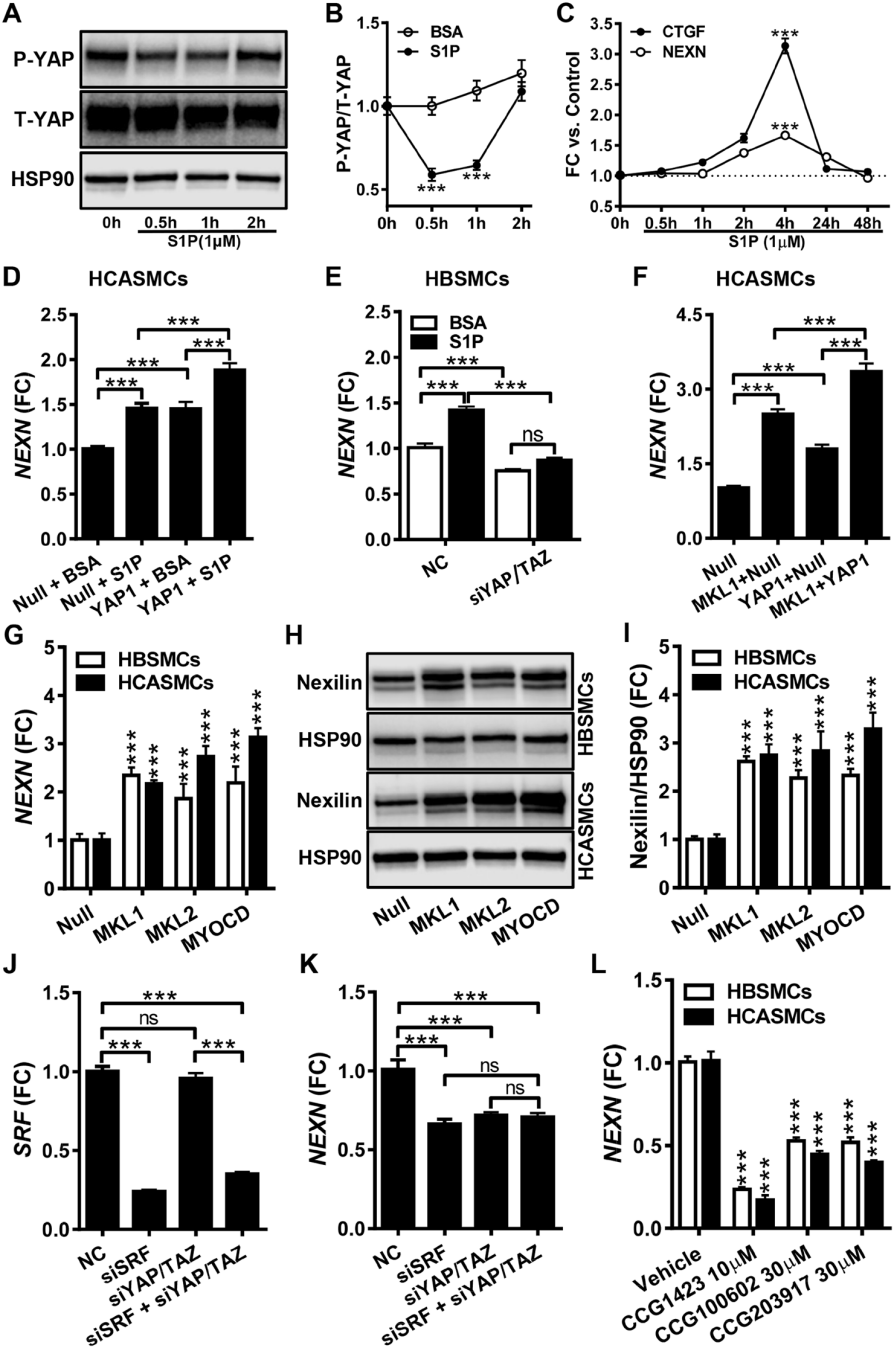
Nexilin/*NEXN* expression is controlled by YAP and by myocardin family coactivators. Panel A shows phosphorylation of YAP (P-YAP) in human coronary artery SMCs (HCASMCs) prior to and at different times after addition of sphingosine-1-phosphate (S1P). Panel B shows compiled data on YAP phosphorylation (N = 9). In panel C, qRT-PCR for *NEXN* and *CTGF* at different times after S1P addition is shown (N = 9). Panel D shows *NEXN* mRNA expression following adenoviral transduction of *YAP1* and S1P treatment, individually and in combination (N = 11). Panel E shows the effect of dual silencing of YAP(*YAP1)* and TAZ (*WWTR1*) on *NEXN* mRNA expression under basal conditions and after S1P stimulation of human bladder SMCs (HBSMCs) (N = 9). Panel F shows the mRNA level of *NEXN* after overexpression of MRTF-A/*MKL1* and *YAP1*, alone and in combination (N = 9). Panel G shows the mRNA level of *NEXN* in control conditions and following overexpression of MRTF-A/*MKL1*, MRTF-B/*MKL2* and myocardin/*MYOCD* (HBSMCs, N = 6–8; HCASMCs, N = 6). Panel H shows western blots for Nexilin following overexpression of MRTFs in bladder (top) and coronary artery (bottom) SMCs, and panel I shows compiled protein data (N = 9). Panel J shows the mRNA level for *SRF* following silencing of *SRF*, YAP/TAZ, or both. Panel K shows the associated reduction of the *NEXN* mRNA (N = 6). Panel L shows the effect of pharmacological inhibition of MRTF/SRF signaling in bladder and coronary artery SMCs (HBSMCs, N = 12; HCASMCs, N = 9).

The F- to G-actin ratio controls nuclear gene activity via MRTFs and YAP/TAZ. Because *YAP1* correlated tightly with *NEXN* across tissues, we focused our initial efforts on this coactivator. One way to activate YAP is to stimulate cells with sphingosine-1-phosphate (S1P)^44^. When SMCs were stimulated with S1P, transient reduction of YAP phosphorylation, and thus activation, was seen in both coronary (Fig. 4A,B) and bladder (not depicted) SMCs. The *NEXN* mRNA level was transiently increased with a peak at 4 h, similar to the archetypal target gene *CTGF* (Fig. 4C). We next overexpressed *YAP1* using an adenovirus, both alone and in combination with S1P treatment. YAP1 and S1P induced *NEXN* in both coronary artery (Fig. 4D) and bladder (not shown) SMCs and their effects were additive.

To further probe the role of YAP1 in *NEXN* regulation we used siRNAs for knockdown of YAP (*YAP1*) and TAZ (*WWTR1*) in bladder SMCs (Fig. 4E). Dual knockdown (*YAP1* by 62.6 ± 4.0% and *WWTR1* by 57.7 ± 4.0%) was done to avoid compensatory induction of TAZ/*WWTR1*, and this led to reduced basal *NEXN* expression, and abrogated induction of *NEXN* by S1P (Fig. 4E). Contrasting with bladder SMCs, YAP1 and TAZ knockdown in coronary artery SMCs did not reduce the basal level of *NEXN*, nor did it mitigate *NEXN* induction by S1P, which rather appeared amplified, but YAP/TAZ knockdown was less effective in these cells (data not shown). We therefore asked if MRTFs, which are controlled by the F-/G-actin ratio and S1P similar to YAP/TAZ^5,45^, communicate changes in actin polymerization to the *NEXN* gene in arterial SMCs. To address this, we transduced coronary artery SMCs with MRTF-A/*MKL1*, both alone and in combination with *YAP1* (Fig. 4F). MRTF-A/*MKL1* appeared more effective than *YAP1* at the same virus titer, and the effects of MRTF-A/*MKL1* and *YAP1* were additive (Fig. 4F). Control experiments showed that eGFP-MKL1 localized to nuclei in some, but not all, transduced cells (not depicted).

Having found that MRTF-A/*MKL1* activates the *NEXN* gene, we next examined if *NEXN* induction occurred with all MRTFs. For this we overexpressed MRTF-A/*MKL1*, MRTF-B/*MKL2* and myocardin/*MYOCD* in side-by-side wells using both bladder and coronary artery SMCs, and analyzed the *NEXN* mRNA and protein levels. All MRTFs increased the *NEXN* mRNA (Fig. 4G) and protein (Fig. 4H,I) in both cell types.

Because knockdown of YAP/TAZ failed to reduce *NEXN* in coronary artery SMCs (see above), and because MRTFs were capable of controlling *NEXN* gene activity similar to *YAP1* (Fig. 4F–I), we next asked if MRTFs contribute to basal *NEXN* expression in SMCs. Combined partial (33–84%, P < 0.001, N = 6) silencing of all MRTFs (MRTF-A, MRTF-B, and MYOCD) did not reduce the *NEXN* mRNA level, but increased TAZ/*WWTR1* (P < 0.001, N = 6, not shown), suggesting compensation. To avoid quadruple knockdown, we instead focused on *SRF* which mediates DNA binding of MRTFs. The siRNA for *SRF* reduced the *SRF* mRNA by 76.1 ± 2.1% and the *NEXN* mRNA by 33.8 ± 7.8% (Fig. 4J,K, HBSMCs). This effect appeared to be non-additive with double knockdown of YAP/TAZ. We also treated bladder and coronary artery SMCs with pharmacological inhibitors of MRTF/SRF signaling (CCG-1423, CCG-100602 and CCG-203971). This reduced *NEXN* by ≥50% in both cell types (Fig. 4L), and the effects tended to be greater in coronary artery SMCs. These experiments, taken together, argue that *NEXN* can be controlled by two families of actin-controlled transcriptional co-activators, YAP/TAZ and the MRTFs. MRTFs appear to contribute more to overall *NEXN* expression, but YAP/TAZ clearly also contribute in bladder SMCs.

### The Nexilin promoter contains CArG-like sequences and is regulated by MRTF-A/MKL1 and MYOCD

**Figure 5:**
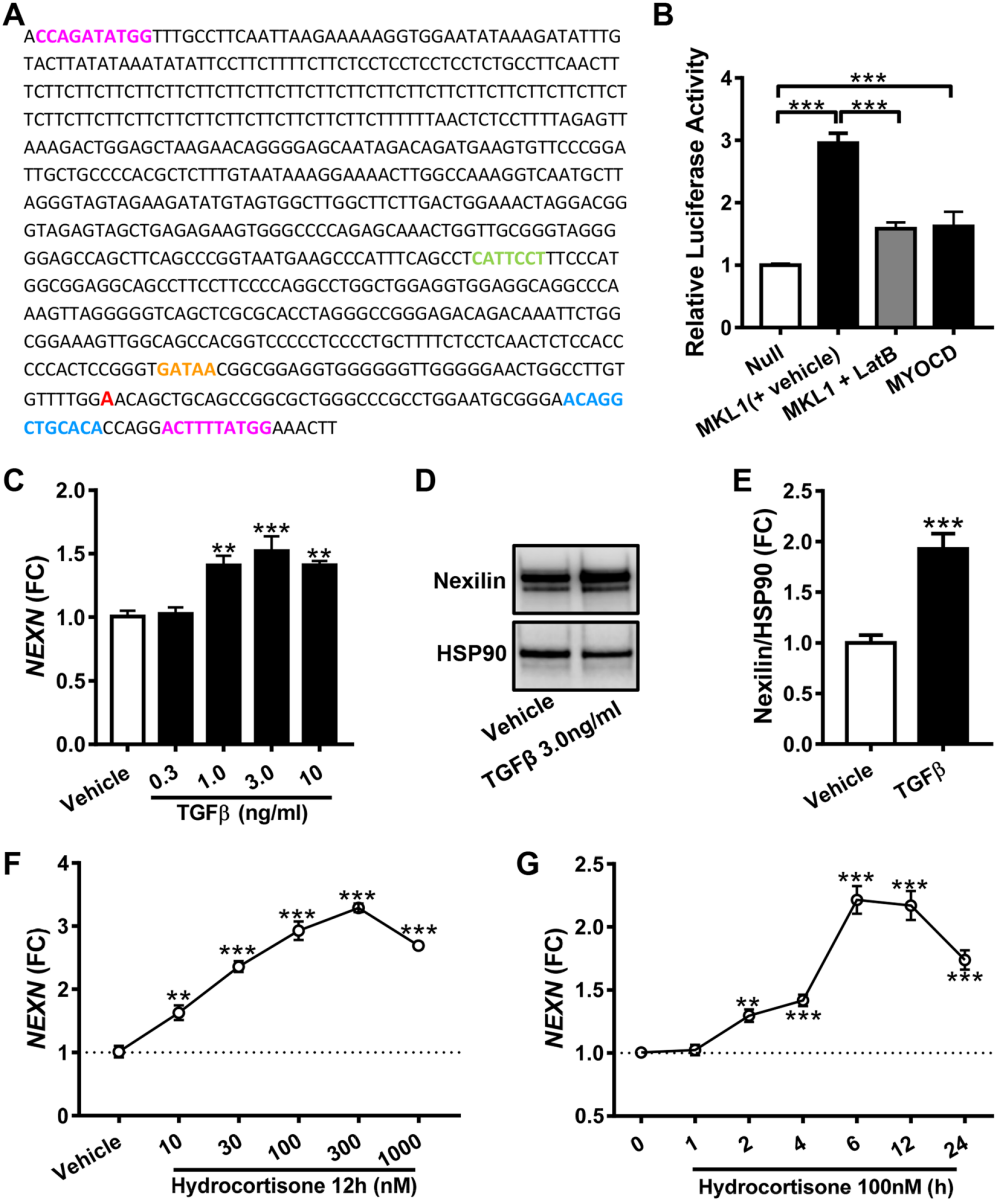
The proximal promoter of *NEXN* is sensitive to myocardin family coactivators. The promoter of *NEXN* (panel A) contains two CArG-like sequences (pink/red), an MCAT motif (i.e. a motif binding YAP/TEAD, green), a GATA motif (orange), and a glucocorticoid receptor motif (blue). Panel B shows reporter activity after transduction of MRTF-A/*MKL1* and myocardin/*MYOCD*, respectively (N = 9). Combined transduction with *MKL1* and LatB treatment, compared to transduction with *MKL1* and treatment with DMSO was done in a separate experiment (N = 6), but is summarized in the same graph for brevity. Panel C shows induction of the *NEXN* mRNA following treatment with TGFβ (N = 6). Corresponding protein data is shown in panels D and E (N = 6). Panels F and G show *NEXN* mRNA expression at different time points (N = 4) and concentrations (N = 8) of hydrocortisone treatment.

SRF acts through genomic elements referred to as CArG boxes (CC(A/T)_6_GG). We surveyed computational predictions^10^ of CArG boxes and found that a CArG-like box in the human *NEXN* promoter had been identified. Manual inspection of a commercial promoter reporter clone (Fig. 5A) showed that it contains the CArG-like box at position +57 (ACTTTTATGG, bottom pink/red motif), and an additional, but poorer, CArG match at position −710 (CCAGATATGG, top pink/red motif). An MCAT-motif that allows for binding of YAP/TEAD was also found at position −232 (CATTCCT, green lettering). This is consistent with ChIP-Seq experiments for TEAD in the Genome Browser (not depicted). A GATA-motif (at −47, orange) and a glucocorticoid receptor motif (at position +40, blue) were also noted. The human *NEXN* promoter thus contains sequence motifs potentially allowing for regulation by actin-controlled coactivators.

To examine whether the *NEXN* promoter can be activated by MRTF-A/*MKL1* and *MYOCD*, the reporter plasmid was transfected into HCASMCs and MRTF-A/*MKL1* and *MYOCD* were overexpressed. Both MRTF-A/*MKL1* and *MYOCD* increased reporter activity (Fig. 5B), and the effect of the former was antagonized by depolymerization of actin using LatB (Fig. 5B). MRTFs and YAP/TAZ are known to be activated downstream of TGFβ. Accordingly, the *NEXN* mRNA (Fig. 5C) and protein levels (Fig. 5D,E) were modestly increased by TGFβ treatment. In view of the glucocorticoid receptor motif in the *NEXN* promoter, we also treated cells with hydrocortisone. A time- (Fig. 5F) and concentration- (Fig. 5G) dependent effect of hydrocortisone was seen on *NEXN* at the mRNA level.

### *NEXN* expression during phenotypic modulation *in vivo*

**Figure 6:**
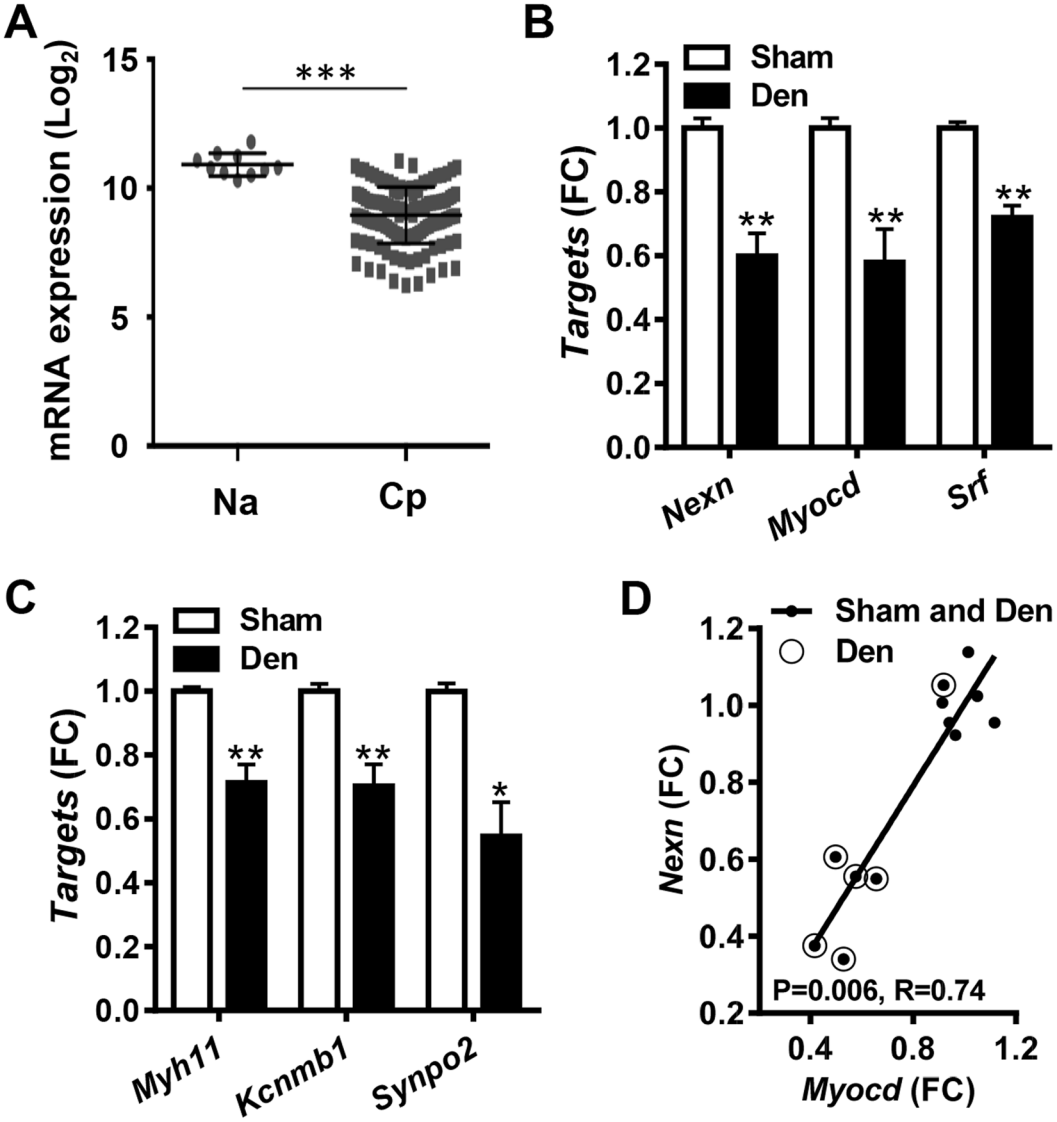
***NEXN*** behaves like an SMC differentiation marker *in vivo*. Panel A shows the Log2 expression level of *NEXN* in normal human arteries (Na, N = 10) and carotid plaques (Cp, N = 127), respectively (data from the Bike database, see Materials and Methods). Panels B and C show expression of *Nexn*, *Myocd* and *Srf* (**B**) along with established SMC markers (**C**) following rat bladder denervation (N = 6 sham-operated control bladders, white bars, and N = 6 denervated bladders, black bars, see Materials and Methods for detail). Panel D shows that *Myocd* and *Nexn* correlate in the bladder dataset (P-value and Spearman Rho coefficient are given in graph).

MRTF/SRF signaling plays a key role in phenotypic modulation of SMCs, such as in atherosclerosis and neointima formation where the expression of MRTF/SRF target genes is reduced. We therefore next asked if *NEXN* is reduced in human atherosclerotic plaques. In keeping with this hypothesis, *NEXN* expression was higher in normal arteries (Na) than in carotid plaques (Cp, Fig. 6A).

We also interrogated a microarray dataset from the denervated rat urinary bladder. Bladder denervation causes considerable hyperplastic detrusor growth, implying phenotypic modulation of SMCs. *Nexn* was reduced in denervated bladders compared to sham controls along with *Myocd* and *Srf* (Fig. 6B). SMC markers, including *Myh11*, *Kcnmb1* and *Synpo2*, were reduced as well (Fig. 6C), and *Myocd* and *Nexn* correlated tightly (Fig. 6D). These findings argued that Nexilin/*NEXN* behaves like an SMC differentiation marker in vascular lesions and in pathological bladder remodeling *in vivo*.

### Nexilin controls actin polymerization in SMCs

**Figure 7:**
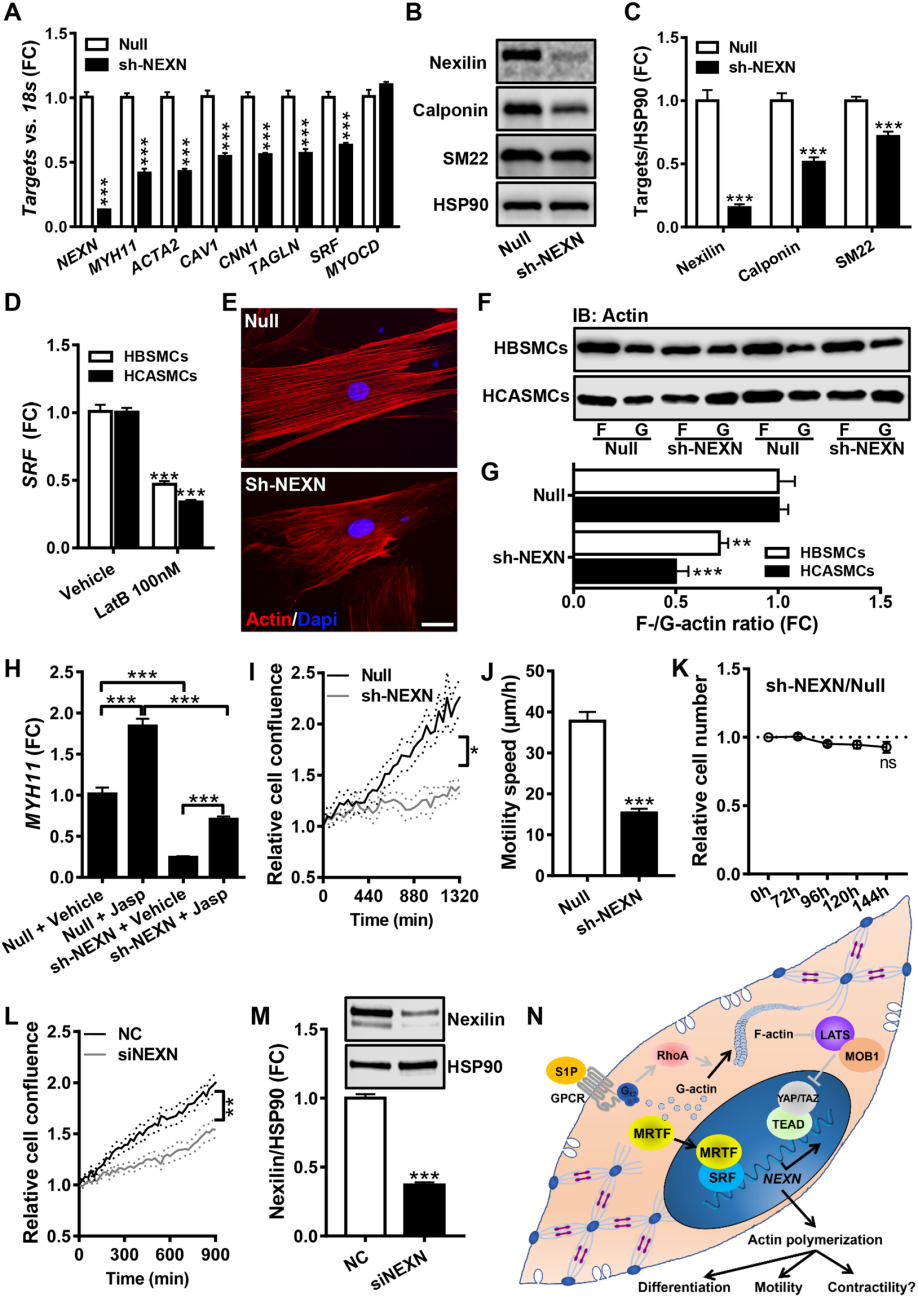
Knockdown of Nexilin/*NEXN* reduces actin polymerization and SMC marker expression. *NEXN* was silenced using a short hairpin adenoviral construct (sh-NEXN) and the contents of *NEXN* and a number of SMC differentiation markers were examined at the mRNA (panel A, N = 6–9) and protein levels (panels B and C, N = 12). Panel D shows that depolymerization of actin using LatB reduces *SRF* expression in both human bladder and coronary artery SMCs (HBSMCs, N = 8; HCASMCs, N = 9). Panel E shows phalloidin staining of actin filaments in control cell (Null) and after *NEXN* silencing (sh-NEXN). The scale bar represents 50 μm. Panel F shows a sedimentation assay to determine filamentous (F-) and globular (G-) actin in control cells (null) and after silencing of *NEXN*. Panel G shows the normalized F- to G-actin ratio in bladder and coronary artery SMCs (HBSMCs, N = 12; HCASMCs, N = 6). Panel H shows that polymerization of actin using jasplakinolide (Jasp) increases *MYH11* in HBSMCs in control conditions and after *NEXN* silencing (N = 6). Panel I shows cell density at different times following the creation of a cell-free area in the culture dish (N = 9–10). Panel J shows the speed of cell movement within the cell-free area (N = 19 and 22 motile cells in Null and sh-*NEXN*, respectively). Panel K shows a cell viability assay comparing control and *NEXN*-silenced cells (N = 12 throughout). The effect of *NEXN* silencing on cell migration was confirmed using an independent siRNA in L (N = 9). The associated repression of the *NEXN* protein is shown in (**M**) (N = 6). The schematic illustration in panel N summarizes our findings regarding the transcriptional control of *NEXN* and its impact on actin polymerization and cell motility. Several aspects of this model, including the involvement of a (**G**) protein-coupled receptor in the S1P effect, were not directly tested here, and they are thus drawn in grey.

Having provided insight into the actin-dependent regulation of the *NEXN* gene, we next addressed Nexilin’s function in SMCs. We used an adenoviral short hairpin construct (Ad-h-*NEXN*-shRNA) for knockdown. We first sought to replicate data in two previous studies^38,39^, showing that *NEXN* manipulation affects SMC differentiation markers. Indeed, smooth muscle myosin (*MYH11*), α-actin (*ACTA2*), calponin (*CNN1*) and caveolin-1 (*CAV1*) were reduced at the mRNA level following *NEXN* knockdown (Fig. 7A). *SRF* was also reduced, but *MYOCD* was unchanged (Fig. 7A). Selected SMC differentiation markers were similarly reduced at the protein level (Fig. 7B,C). Dense bodies and dense bands are tethering points for both contractile and cytoskeletal actin in SMCs, raising the possibility that *NEXN* knockdown may affect actin stability, and indirectly influence differentiation markers via MRTFs. In keeping with this possibility we found that LatB treatment, which depolymerizes actin and reduces differentiation markers, reduced SRF similar to silencing of *NEXN* (Fig. 7D). To examine actin polymerization directly, we stained F-actin using phalloidin. Phalloidin staining was less bright after *NEXN* silencing (Fig. 7E), and filaments appeared somewhat more slender, but this was difficult to quantitate. We therefore instead measured the F/G-actin ratio using a sedimentation assay. *NEXN* knockdown reduced the F/G-actin ratio in human bladder and coronary artery SMCs (Fig. 7F, summarized data in panel G). We also examined if *NEXN* expression was important for expression of differentiation markers following polymerization of actin using jasplakinolide (Jasp). The archetypal SMC differentiation marker *MYH11* was induced by Jasp in control- and in *NEXN* knockdown cells (Fig. 6G), but overall expression levels were lower following *NEXN* knockdown. This argues that *NEXN* is important but not critical for actin-dependent control of gene expression.

We finally examined the effect of *NEXN* silencing on cell coverage of a cell-free area caused by a scratch through the cell monolayer *in vitro*. The cell density in the cell-free area increased over time, as seen using real-time imaging, and this was slower following *NEXN* silencing (Fig. 7I,J). This effect could not be ascribed to altered cell proliferation, because no cell divisions were observed in the cell-free area in any of the experiments. A cell viability assay moreover failed to disclose an effect on relative cell numbers (Fig. 7K), again arguing that the effect was due to impaired cell migration. We confirmed the effect on cell migration using a *NEXN* siRNA targeting a distinct nucleotide sequence in the mRNA than the shRNA used above (Fig. 7L). The siRNA cause almost comparable, but more transient, Nexilin repression (Fig. 7M). The siRNA approach seemed to dissociate the effect on cell migration from the effect on cell differentiation, because SMC markers were reduced by 96 h after siRNA treatment but not earlier, and yet cell migration was reduced already at 72 h (not depicted).

## Discussion

The expression of many SMC-specific genes, as well as a cadre of genes in striated muscles, is controlled by myocardin-related transcription factors (MRTFs). Here we focused on *NEXN* (Nexilin), which is expressed in both striated and smooth muscle. We demonstrate that this gene is regulated by changes in actin polymerization as well as by MRTFs and YAP/TAZ, two families of actin-controlled transcriptional coactivators. Nexilin appears to be localized to dense bands and dense bodies in SMCs, i.e. the functional equivalents of Z-discs in striated muscle, and we find that Nexilin affects the F/G-actin ratio and cell migration. This may be due to an effect on dense body attachment sites for actin. Disturbed actin polymerization is expected to influence SMC marker expression via MRTFs, and we confirm that *NEXN* knockdown has this effect. We therefore propose that Nexilin is a dense body-associated protein that is important for SMC motility and gene expression (see summary graph in Fig. 7N).

*NEXN* mutations have been found to cause cardiomyopathy^34–36^. Whether *NEXN*-dependent cardiomyopathy is defined by dilation or hypertrophy depends on the localization of the *NEXN* mutation. Potentially, the force-transducing and actin-organizing functions of Nexilin localize to different domains of the protein. We have not addressed if *NEXN* is controlled by myocardin in cardiomyocytes, but heart-specific deletion of myocardin gives rise to dilated cardiomyopathy^25^ similar to knockout of *NEXN*^35^. It is therefore tempting to speculate that myocardin-driven *NEXN* expression contributes to Z-disc stability and heart function. Our findings do not support regulation of *NEXN* by myocardin in skeletal muscle because no correlation was observed at the mRNA level in this tissue. This does not rule out a regulatory role of either MRTF-A/MRTF-B or YAP/TAZ. Several MRTF target genes beyond *NEXN* localize to Z-discs/dense bodies/dense bands, including α-actinin^25^ and synaptopodin 2 (SYNPO2, a.k.a. myopodin or fesselin)^46,47^, arguing that MRTFs may be critical for formation of these structures.

Similar to the MRTFs, YAP and TAZ are actin-controlled and mechanoresponsive^48^ coactivators. YAP and TAZ act via TEAD transcription factors, and we found that both *YAP1* and *TEAD1* correlate with *NEXN* across tissues. An emerging picture is that certain targets of YAP/TAZ and MRTFs overlap, and *NEXN* is an example of this. Recent work has indicated that interaction exists between YAP/TAZ and the MRTFs that extends beyond partial overlap of target genes, and involves direct physical binding. For instance, MRTF-A and TAZ may interact via a WW domain-dependent mechanism^13^, and this was shown to be critical for TGFβ induction of SMC target genes^49^. Given that TEAD-binding motifs are present in the *NEXN* promoter such a mechanism may be considered for the induction of *NEXN* by TGFβ. A complex consisting of YAP1, TEAD and NCOA3 has also been shown to confer DNA binding ability on MRTFs^11^, and early work indicated a productive interaction between NCOA3 and myocardin^50^. We note that *NCOA3* correlates tightly across tissues with *NEXN*, suggesting involvement in *NEXN* regulation.

Our results suggest that the transcriptional drive on *NEXN* may differ somewhat between SMCs from different anatomical locations. In bladder SMCs, knockdown of YAP and TAZ reduced basal *NEXN* expression as well as the S1P-stimulated level. In coronary artery SMCs, on the other hand, the basal *NEXN* level was not significantly reduced by YAP/TAZ silencing, and the S1P stimulated level appeared higher. It should be noted however that silencing was less effective in arterial SMCs. We cannot presently explain the apparent discrepancy between urogenital and arterial SMCs, but it is possible that the relationship between the coactivator families differ due to binding partners or epigenetic factors. Of further interest in this regard is our finding that YAP appears to localize together with Nexilin at the membrane of bladder SMCs. Localization of YAP at dense bands has not been reported for vascular SMCs, suggesting one difference between cell types. Z-discs have been proposed to constitute a key component of the stretch sensor machinery in cardiomyocytes^26^, and a similar role of dense bodies/dense bands in certain SMCs is worth considering.

Regulation of *NEXN* by MRTFs may represent a mechanism of two previously described regulatory influences on *NEXN* expression. The first involves *NEXN* mRNA reduction by platelet-derived growth factor (PDGF)^37,38^. PDGF causes phenotypic modulation of SMCs toward the synthetic phenotype. This involves ternary complex factor activation, and resultant competition with MRTFs for SRF binding^8^. The second regulatory influence involves hepatocyte growth factor (HGF) which induces *NEXN* in cardiomyocytes^51^. It has been shown that myocardin is increased by HGF^52^. It thus seems likely that both PDGF and HGF may converge, at least in part, on MRTF/SRF signaling to cause changes in Nexilin/*NEXN* expression.

The notion that single nucleotide polymorphisms in the vicinity of the *NEXN* gene associate with coronary artery disease^38^ is appealing. This was proposed to involve SMC phenotypic switching. Our work confirms that *NEXN* knockdown affects several SMC differentiation markers, and shows that *NEXN* is reduced in the remodeling urinary bladder and in human atherosclerotic plaques. The latter finding is to be expected, because myocardin expression is reduced in vascular lesions, including atherosclerotic plaques, along with numerous SMC differentiation markers^53,54^. A putative mechanism to explain the effect of *NEXN* on SMC marker expression is via changes in actin polymerization, but the exact way by which *NEXN* influences actin polymerization remains to be established. In contrast to prior work^38,39^, we observed no direct effect of *NEXN* silencing on myocardin mRNA expression. We envision that MRTF activation and increased *NEXN* expression would favor an increased F- to G-actin ratio which would then further promote MRTF activation in feed forward manner. *NEXN* is not the first MRTF target engaged in such amplification of SMC differentiation. A similar model was recently proposed for the ion channel TMEM16A/*ANO1*^17^, and another example is the long non-coding RNA MYOSLID^55^.

When examining the *NEXN* promoter we observed sequence motifs suggesting regulation by glucocorticoids, and we were able to confirm this experimentally. The broader implications of this control mechanism are unknown. It may however allow for an impact of glucocorticoids on phenotypic modulation of SMCs. If *NEXN* is similarly responsive to glucocorticoids in cardiomyocytes remains to be established, but this could have therapeutic implications in heart disease caused by partial loss-of-function mutations in *NEXN*.

To summarize, our findings suggest that Nexilin/*NEXN* is a dense body/dense band-associated protein in SMCs and show that it promotes actin polymerization and amplifies SMC differentiation. Myocardin family coactivators and YAP/TAZ, which represent two families of actin-regulated coactivators, control *NEXN* expression via sequences in the proximal promoter. *NEXN* is therefore endowed with the dual ability to control and respond to changes in the F- to G-actin ratio.

## Material and Methods

### Primary human cells and tissues

Human bladder tissue from six individuals was excised after surgery as described^56,57^. Informed consent was obtained in writing, and the protocol was approved by the Ethical Review Board at Lund University (http://www.epn.se, approval number 2008-4). All methods were performed in accordance with this approval and with relevant guidelines and regulations. Detrusor strips were prepared by microdissection, and cells were isolated from strips from four individuals. Strips from four separate individuals were used for immunostaining. For isolation of human bladder SMCs (HBSMCs), muscle strips were cut into fine pieces and incubated in serum-free Dulbecco’s Modified Eagle’s Medium (DMEM, Gibco, 11966-025) containing 2 mg/ml collagenase type-2 (Worthington Biochemical Corporation, LS004176) and 0.2 mg/ml elastase (Sigma, E7885, at 37 °C in a humidified atmosphere of 95% air/5% CO_2_) for 3 h with gentle tumbling every 30 min. After sedimentation of debris, the supernatant was transferred to a sterile 15 ml tube and centrifuged (1000RPM, 3 min). The cell pellet was then washed using Phosphate Buffered Saline (PBS, VWR, L1825), and suspended in DMEM/Ham’s F-12 medium with glutamine (Biochrom, FG4815), 10% Fetal Bovine Serum (FBS, Biochrom, S0115), and 50U/50 μg/ml penicillin/streptomycin (Biochrom, A2212). Human coronary artery SMCs (HCASMCs) were from Gibco (Life Technologies, 1130140), and were cultured in Medium 231 (Life Technologies, M231500) with 5% growth supplement (SMGS, Life Technologies, S-007-25) and 50U/50 μg/ml penicillin/streptomycin. Cells were cultured in a water-jacketed cell culture incubator and media were refreshed every 48 h. Cells were passaged by trypsin (Gibco, 25200056) treatment and used between passages 3 and 8.

Latrunculin B (LatB), used here to cause depolymerization of actin, is inactivated by serum. Thus, for treatment of HBSMCs with LatB (Calbiochem, #76343-94-7), cells were washed twice and incubated in 0% FBS DMEM/Ham’s F-12 medium 24 h after seeding. 300 nM of LatB was then added and cells were harvested 24 h later. For concentration-response relationships, cells were seeded and washed as described above and then treated with 10, 30, 100, 300 and 1000 nM of LatB. For treatment of HCASMCs with LatB, cells were washed twice with 0% SMGS Medium 231 24 h after seeding. 100 nM of LatB was then added to 0% SMGS Medium 231 and maintained for 24 h. For polymerization of actin, cells were treated with 100 nM jasplakinolide (Tocris Bioscience, 2792) for 96 h. The equivalent concentration of DMSO (Sigma Aldrich, D5879) was used as vehicle control in all of the experiments above.

For treatment with inhibitors of MRTF/SRF signaling, cells were moved to serum-free medium 24 h after seeding, followed by addition of CCG-1423 (10 µM, Calbiochem, 555558), CCG-100602 (30 µM, Cayman chemical, 10787), CCG-203971 (30 µM, Cayman chemical, 15075), or the corresponding concentration of DMSO. Cells were harvested 24 h later. For treatment with hydrocortisone, HBSMCs were washed twice with 0% FBS DMEM/Ham’s F-12 medium 24 h after seeding, and 100 nM of hydrocortisone (Sigma, H4001) or the same volume of DMSO were added and kept for the times indicated. For concentration-response experiments, 10, 30, 100, 300, 1000 nM of hydrocortisone, or the corresponding concentration of DMSO, were added and maintained for 12 h.

For treatment with transforming growth factor β (TGFβ1, R&D systems, 240-B), HBSMCs were incubated with 0.3, 1.0, 3.0 or 10 ng/ml of TGFβ1 or vehicle (4 mM HCl containing 1 mg/mL bovine serum albumin (BSA, Sigma, A3059)) for 48 h in serum-free medium. Sphingosine-1-phosphate (S1P, Sigma-Aldrich, S9666) was prepared and aliquoted according to the manufacturer’s instruction, and added to HBSMCs and HCASMCs (1 µM) 24 h after seeding and in serum-free medium. Cells were then harvested at the indicated time points.

### *NEXN* mRNA expression in human tissues

RNA-Seq data was downloaded from the Genotype-Tissue Expression (GTEx) project (http://www.gtexportal.org/)^40^ in Aug, 2016. The trimmed mean of M-values (TMM) normalization method was applied^58^. Individual correlation analyses were done using the Spearman method in GraphPad Prism. In extended analyses, *NEXN* was correlated with all other transcripts in the ten tissues with highest expression levels using Pearson correlation analysis in Excel. The sum of correlation coefficients for individual tissue RNAs (R_sum_) was then calculated and sorted in descending order. Selected correlations from the extreme of the R_sum_ distribution were tested using the Spearman method. The number of individuals varied depending on the tissue, and the range was between 83 and 430 with a median of 206. For the coronary artery, which was used as an example, data was available from 133 individuals.

### Staining for Nexilin/*NEXN*

Immunohistochemistry for *NEXN* (HPA011185) was inspected in the Human Protein Atlas^41^, and examples from heart, skeletal muscle, esophagus and gall bladder were chosen for visualization of SMC-enrichment of *NEXN*. To confirm *NEXN* expression in SMCs we used cryo-sections of fresh-frozen human bladder detrusor strips. Fresh muscle strips were embedded in O.C.T. COMPOUND (VWR, 361603E) and frozen, and 10 µm cross-sections were cut. Sections were blocked with 3% BSA for 60 min at room temperature before incubation with the following primary antibodies overnight: Nexilin (1:200 in 3% BSA, Abcam, ab213628 or HPA011185, Sigma), CAV1 (1:400 in 3% BSA, Cell Signaling Technology, #3267) and YAP1 (1:200 in 3% BSA, Cell Signaling Technology, #4912). After washing, the slides were incubated with Goat anti-Mouse IgG Alexa Fluor Plus 488 (1:200, Invitrogen, A32723) and donkey anti-rabbit Alexa Fluor® 647 (1:200, Invitrogen, A31573). A goat anti-rabbit Alexa Fluor® 488 (1:200, Invitrogen, A11008) secondary antibody was used for detecting Nexilin (HPA011185, Sigma) or YAP1 before co-staining with direct-conjugated CAV1 antibody, which was a kind gift from Dr. Karin Stenkula. Nuclei were counterstained with DAPI (Invitrogen, D1306). The slides were mounted with Fluorescence Mounting Medium (Dako, S3023). Images were taken using an Olympus DP72 microscope equipped with a digital camera. The software Olympus CellSens Dimension was used for image capture. Nexilin, CAV1 and YAP1 were imaged using a laser scanning confocal microscope (LSM5, PASCAL, Carl Zeiss AG).

### Immuno-electron microscopy

Human bladder strips were fixed in 0.1 M PBS containing 1.5% paraformaldehyde and 0.5% glutaraldehyde. Specimens were dehydrated in ethanol and infiltrated in Lowicryl HM20 which was subsequently polymerized using UV light at −50 °C. 50 nm thick sections were cut in a Leica Ultrotom UC7 and mounted on pioloform-coated gold grids (Maxtaform H5). For immunostaining, sections were blocked with 1% BSA in PBS followed by incubation with Nexilin antibody (1:80-1:200, Abcam, ab213628) in a damp chamber at 4 °C. Following washing in PBS, grids were incubated with a secondary antibody conjugated with 10 nm gold. After further washing in PBS, sections were contrasted in 4% uranylacetate at 40 °C. Images were captured in a FEI Tecnai 120kv Biotwin electron microscope. Out of focus imaging was used for unambiguous identification of gold particles. The final density of gold particles was too low for random image acquisition.

### Adenoviral treatment

Adenoviruses for overexpression of *YAP1* (Ad-h-YAP1, ADV-227945), MRTF-A (*MKL1*, Ad-h-MKL1/eGFP, ADV-215499), MRTF-B (*MKL2*, Ad-h-MKL2, ADV-215500) and *MYOCD* (Ad-h-MYOCD, ADV-216227) were obtained from Vector Biolabs. Empty virus vector (Ad-CMV-Null, Vector Biolabs, 1300) at the same multiplicity of infection (MOI) was used as control. For overexpression of *MKL1*, *MKL2* and *MYOCD*, HBSMCs and HCASMCs were transduced with 100 MOI 24 h after seeding. Cells were maintained in virus-containing medium for 24 h, and for another 72 h in fresh medium before harvest. For double overexpression of *MKL1* and *YAP1*, cells were transduced with 200 MOI CMV-Null, or with 100 MOI Ad-h-MKL1 plus 100 MOI CMV-Null, or with 100 MOI Ad-h-YAP1 plus 100 MOI CMV-Null, or with 100 MOI Ad-h-MKL1 plus 100 MOI Ad-h-YAP1. For overexpression of *YAP1*, HCASMCs were transduced with 100 MOI Ad-h-YAP1 or CMV-Null 24 h after seeding. Cells were then maintained in virus-containing medium for 24 h, and for another 48 h in fresh medium. S1P was then finally added for 4 h as described above.

For knockdown of Nexilin/*NEXN* we used a short hairpin construct (Ad-GFP-U6-h-*NEXN*-shRNA, shADV-230496) and compared this with a null construct (Ad-GFP-U6-shRNA, Vector Biolabs, 1122) as control. 24 h after seeding, HBSMCs or HCASMCs were transduced with 300 MOI of Ad-GFP-U6-h-*NEXN*-shRNA or control virus, respectively. Cells were maintained in virus-containing medium for 24 h, and for another 96 h in fresh medium. Some of these cells were then treated with jasplakinolide before harvest.

### siRNA-mediated knockdown of YAP/TAZ, SRF and *NEXN*

The following Silencer® Select Pre-designed siRNAs were purchased from Ambion Thermo Scientific: *YAP1* (s534571), *WWTR1* (s24788), *SRF* (s13427), *NEXN* (s228462), and Negative Control siRNA (AM4635). The *NEXN* siRNA is predicted to bind 605 bases from the translation start codon whereas the *NEXN* shRNA used (see above) targets a sequence 1508 bases from the start codon. SMCs were cultured as described above. 24 h after seeding, cells were transfected with 50 nM *SRF* siRNA, 50 nM *YAP1* siRNA plus 50 nM *WWTR1* siRNA, or 50 nM *SRF* siRNA plus 50 nM *YAP1* siRNA plus 50 nM *WWTR1* siRNA using Oligofectamine transfection reagent (Invitrogen, 12252-011) and Opti-MEM I reduced serum medium (Gibco, 11058021). The silencing of *NEXN* using siRNA is described under Cell Migration below. Negative Control siRNA was used to balance the total amount of siRNA. Cells were harvest or treated with S1P as described above 72 h after transfection. Targeted genes were typically reduced by more than 50% after siRNA transfection compared to the negative control.

### RNA extraction and RT-qPCR

After treatment, HBSMCs and HCASMCs were washed twice with ice-cold PBS and lysed in Qiazol (Qiagen, Cat. 79306). Total RNA was extracted with the Qiagen miRNeasy mini kit (Qiagen, 217004), and concentration and purity were determined using the Nanodrop 2000c spectrophotometer (Thermo Scientific). Reverse transcription qPCR reactions were prepared using the Quantifast SYBR Green RT-PCR kit (Qiagen, 204156) with Quantitect (Qiagen) primer assays for *ACTA2* (QT00088102), *CAV1* (QT00012607), *CNN1* (QT00067718), *CTGF* (QT00052899), *MYH11* (QT00069391), *MYOCD* (QT00072884), *NEXN* (QT00006251), *SRF* (QT00084063), *TAGLN* (QT00072247), and *18 S* (QT00199367). The latter was used as a reference gene throughout. The validated primer sequences are not disclosed by Qiagen. RT-qPCR reactions were run using the StepOnePlus qPCR cycler (Applied Biosystems).

### Western blotting

Cultured SMCs were washed with ice cold PBS twice, and 80 μl Laemmli sample buffer (60 mM Tris-Hcl, pH 6.8, 10% glycerol, 2% SDS) was added. After lysis and protein determination using a detergent-compatible protein assay from Bio-Rad (500–0116), bromophenol blue (0.01%) and β-mercaptoethanol (5%) were added to the remainder of the lysates. ≈20 μg protein was loaded per lane on Bio-Rad TGX Criterion gels and proteins were separated using Bio-Rad Tris/Glycine/SDS electrophoresis buffer at 200 V. Following separation, proteins were transferred to nitrocellulose (0.2 μm) using the Trans-Blot Turbo Transfer System (Bio-Rad) and western blotting was done essentially as described^57^. Membranes were cut horizontally to allow for detection of multiple targets as needed, and hence blots covering the entire range of molecular weights are not available throughout. Full blots for all display items are available in the Supplementary Information file. The following primary antibodies were used: *NEXN* (Abcam, ab213628), P-YAP (Cell Signaling Technology,#4911), T-YAP (Cell Signaling Technology, #4912), HSP90 (BD Transduction Laboratories, 610418), calponin/*CNN1* (Abcam, ab46794), SM22/*TAGLN* (Abcam, ab14106). Secondary anti-mouse and anti-rabbit antibodies were from Cell Signaling Technology (#7076 S, #7074 S).

### Promoter reporter assays

HCASMCs were seeded in 6-well plates and transduced with adenovirus as described above. 24 h after transduction, cells were transfected with promoter reporter plasmid for *NEXN* (GeneCopeia, HPRM37365-PG04, containing both Gaussia Luciferase and secreted alkaline phosphatase) using FuGENE® 6 Transfection Reagent (Promega, E2691). 100 μl medium was collected 72 h after transfection and stored at −20 °C. For LatB treatment, cells were treated 24 h after transfection and 100 μl medium was collected after 48 h of LatB incubation. Promotor reporter activity was measured using a dual luciferase kit and high-sensitivity GL-H buffer (Secrete-Pair Dual Luminescence Assay Kit, Genecopeia) according to the manufacturer’s instructions. Briefly, 10 μl medium was mixed with substrate and incubated for 30 s before measurement using a Glomax Luminometer (Promega). Readings were normalized to secreted alkaline phosphatase readings.

### *NEXN* expression in phenotypic modulation *in vivo*

To explore if *NEXN* expression was affected in human atherosclerotic plaques compared to healthy human arteries, we used the BiKE (Biobank of Karolinska Endarterectomy) database^59–61^. In BiKE, gene expression analyses using Affymetrix HG-U133 2.0 arrays are available for carotid atheromas from 127 patients and 10 normal arteries from organ donors. Log2 mRNA expression level were plotted and used for statistical testing. The microarray dataset is available at the Gene Expression Omnibus (GSE21545).

Bladder denervation causes sizeable growth (>5-fold) of detrusor smooth muscle implying phenotypic modulation. In recent work we charted gene expression changes in the rat urinary bladder at 10 days following freezing of pelvic ganglia^56^, and this dataset (available at the Gene Expression Omnibus, GSE104540) was used here.

### Staining for F-actin

HBSMCs were seeded in a chamber slide (SPL Lifesciences, 30108) at a density of 2000 cells/well. 24 h after seeding, cells were transduced with adenoviruses for knockdown of *NEXN* (300MOI) or the equal amount of null construct as control. Staining for F-actin was performed 120 h after transduction. Briefly, after washing with PBS, cells were fixed in 4% paraformaldehyde for 15 min at room temperature, followed by a permeabilization step with 0.25% Triton-X for 20 min at room temperature. Cells were then blocked with 3% BSA for 60 min at room temperature before incubation with the Alexa Fluor™ 633 Phalloidin (1:200, Invitrogen, A22284) for 50 min. Then the chamber walls were removed. Nuclei were counterstained with DAPI, and slides were mounted with Fluorescence Mounting Medium as describe above.

### Actin polymerization

Actin polymerization was measured using a protocol adapted from Rasmussen *et al*.^62^. Control and *NEXN* knockdown SMCs were washed twice in PBS and lysed in 100 μl actin-stabilizing lysis buffer (containing 0.1 M PIPES (pH 6.9), 30% glycerol, 5% DMSO, 1 mM MgSO_4_, 1 mM EGTA, 1% Triton-X100, 1 mM ATP, and protease inhibitor cocktail (Sigma-Aldrich, P8340)). Lysates were transferred to 1.5 ml Eppendorf tubes and centrifuged for 75 min at 20000 G at 4 °C. Supernatants were transferred to new tubes and pellets remaining in the original tubes were dissolved in actin depolymerizing buffer (0.1 M PIPES (pH 6.9), 1 mM MgSO_4_, 10 mM CaCl_2_ and 5 μM cytochalasin D) by sonication. Samples were then prepared from the supernatants (G-actin) and dissolved pellets (F-actin) by mixing with Laemli sample buffer. SDS-PAGE and western blotting was done as described above using a pan-actin antibody (#AAN01, Cytoskeleton Inc.). F- and G-actin samples for the different treatment groups were loaded next to each other. After densitometric scanning in Image Studio, the F/G-actin ratio was calculated by dividing the signals for the F- and the G-actin lanes, respectively.

### Cell migration

Cell migration was measured using a scratch assay, and the cell confluence as well as the speed of the cell motility in the wound were followed over time using a real-time cell imaging system (HoloMonitor M4, Phase Holographic Imaging, Lund Sweden). HBSMCs were seeded in 6-well plates (Sarstedt, 83.3920.005). 24 h after seeding, cells were transduced with 300 MOI of Ad-GFP-U6-h-*NEXN*-shRNA or control virus, respectively. After another 24 h, the virus-containing media were exchanged for fresh media with 10% FBS to establish cell confluence. 12 h before the assay started, FBS was omitted from the medium. A scratch was made using a Gelloader Pipette Tip (Sarstedt, 70.1190.100), and the cell-free area was imaged every 40 min to monitor cell motility, cell divisions, and cell confluence. Images were automatically acquired at 40 min intervals for the duration of the experiment (22 h) and stored by the HoloMonitor software system. For *NEXN* silencing using siRNA, HBSMCs were seeded in 6-well plates as above. 24 h after seeding, cells were transfected with 50 nM *NEXN* siRNA and 50 nM Negative Control siRNA, respectively. Cells were then incubated in the siRNA transfection medium for 24 h, followed by a 24 h incubation in DMEM/Ham’s F-12 medium with 10% FBS. 48 h after the first siRNA transfection, another siRNA transfection was done using DMEM/Ham’s F-12 medium with 0% FBS. 24 h after the second siRNA transfection a scratch was made, and images were acquired at 20 min intervals for the duration of the experiment (15 h) in the HoloMonitor as described above. Cell confluence and motility was analyzed using Hstudio M4.

### Cell proliferation

HBSMCs were seeded at a density of 1000 cells/well in 96-well plates. At 24 h, cells were treated with adenoviruses for knockdown of *NEXN* (300MOI). An equal amount of null construct was used as control. Cell numbers at the indicated time points were measured using a Cell Counting Kit-8 (CCK-8, Dojindo, CK04–13) according to the manufacturer’s instructions. Briefly, medium was replaced by 110 μl fresh cell culture medium mixed with 10 μl CCK-8 solution. Plates were then returned to the incubator for 2 h. The absorbance was monitored at 450 nm in a Multiscan GO Microplate Spectrophotometer (Thermo Fisher Scientific).

### Statistical analyses

Statistical calculations were made in Graph Pad Prism. Means ± S.E.M. are plotted in all graphs. Single comparisons were made using a two-tailed student’s t-test. Multiple comparisons were made using one-way ANOVA followed by Bonferroni’s post-hoc test. Cell density changes over time were tested using RMANOVA. Correlations were tested using the Spearman method. P < 0.05 was considered significant. *P < 0.05, **P < 0.01, ***P < 0.001, and ****P < 0.0001.

## Electronic supplementary material

Supplementary Information

## Data Availability

The authors confirm that the data supporting the findings of this study are available within the article and its supplementary materials.
