# Supplementary material for: Nexilin/NEXN controls actin polymerization in smooth muscle and is regulated by myocardin family coactivators and YAP
Source: Sci Rep. 2018 Aug 29;8:13025. doi: 10.1038/s41598-018-31328-2 (PMC6115340; doi:10.1038/s41598-018-31328-2)
Supplement: Supplementary file 1 — Supplementary Information [file 41598_2018_31328_MOESM1_ESM.pdf]

Supplementary information

Nexilin/*NEXN* controls actin polymerization in smooth muscle and is regulated by myocardin family coactivators and YAP

Baoyi Zhu*, Catarina Rippe, Johan Holmberg, Shaohua Zeng, Ljubica Perisic, Sebastian Albinsson, Ulf Hedin, Bengt Uvelius, Karl Swärd

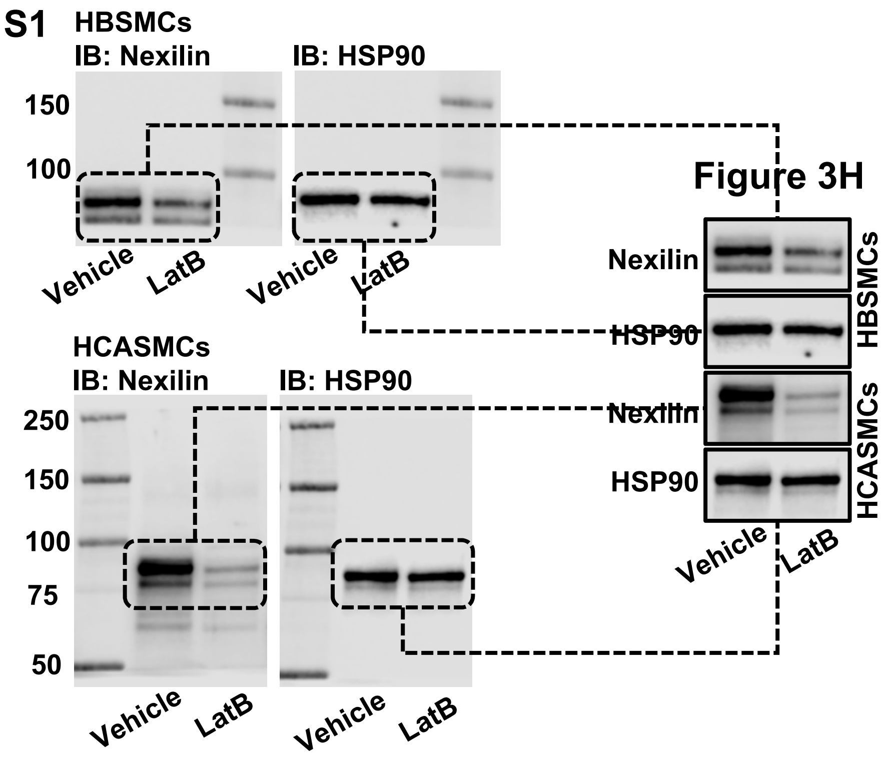

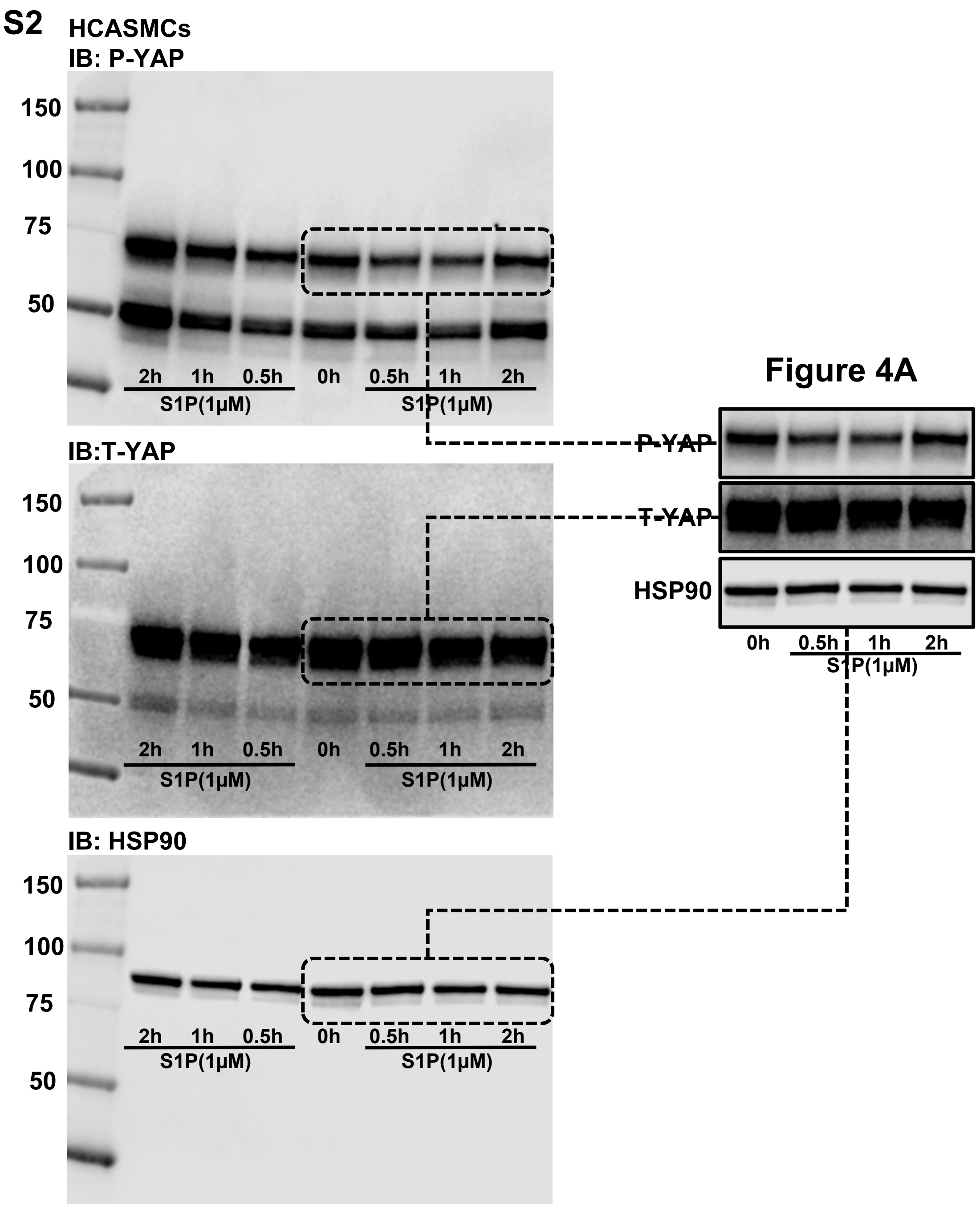
S2
HCASMCs
IB: P-YAP
150
100
75
50
2h
1h
0.5h
0h
0.5h
1h
2h
S1P(1μM)
S1P(1μM)
IB:T-YAP
IB: HSP90
Figure 4A
P-YAP
T-YAP
HSP90

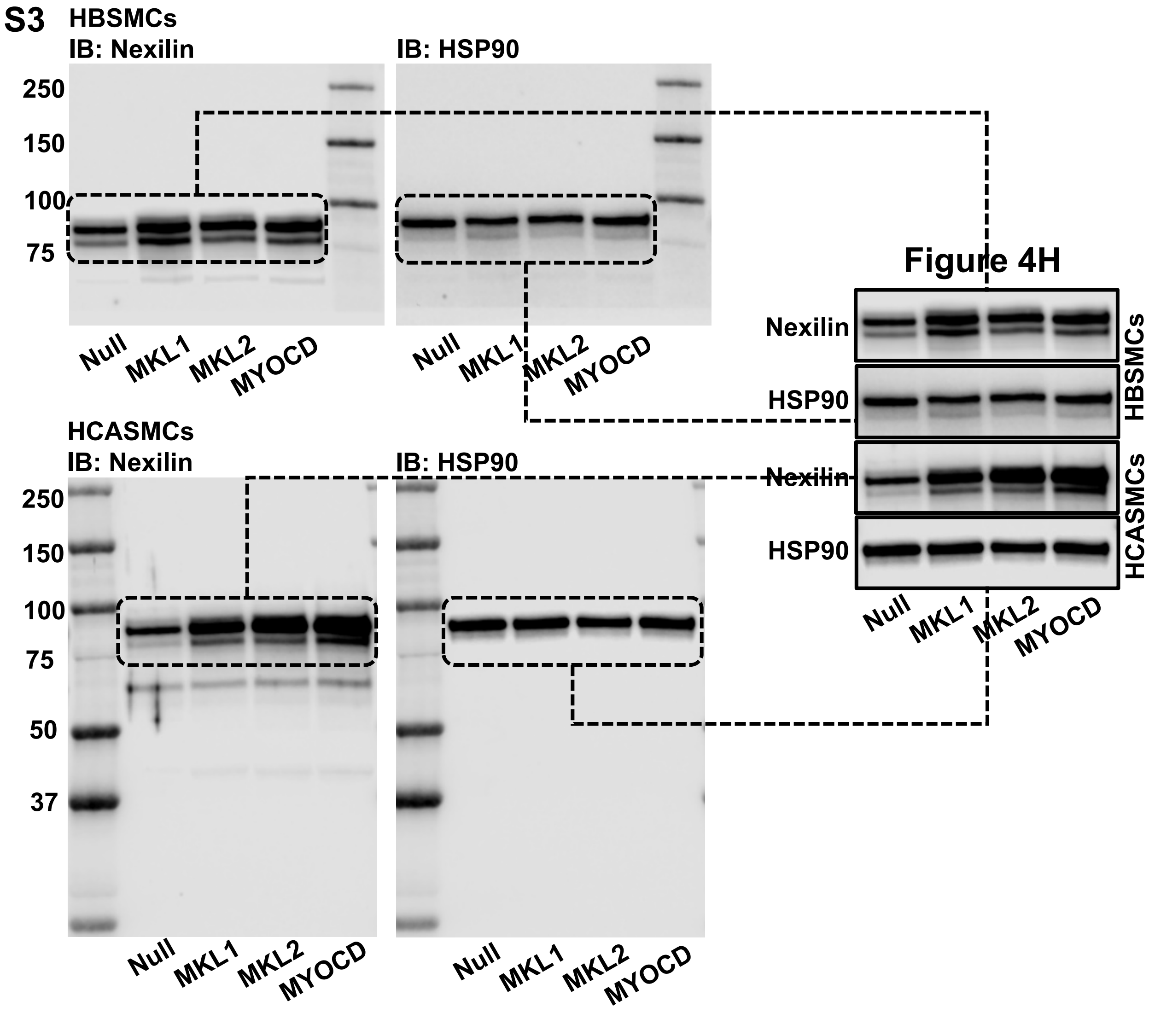
S3
HBSMCs
IB: Nexilin
IB: HSP90
250
150
100
75
Null
MKL1
MKL2
MYOCD
HCASMCs
IB: Nexilin
IB: HSP90
250
150
100
75
50
37
Figure 4H
Nexilin
HSP90
HBSMCs
HCASMCs

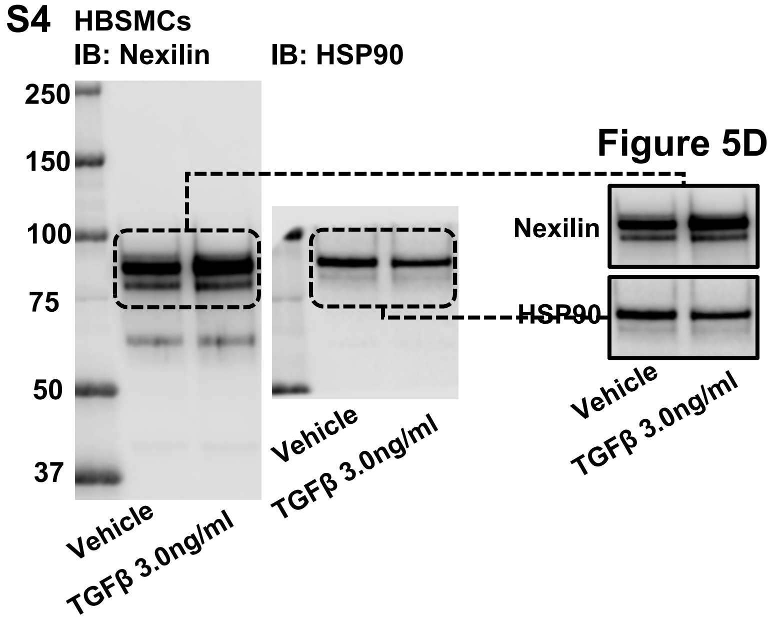
S4
HBSMCs
IB: Nexilin
IB: HSP90
250
150
100
75
50
37
Figure 5D
Nexilin
HSP90
Vehicle
TGFβ 3.0ng/ml
Vehicle
TGFβ 3.0ng/ml
Vehicle
TGFβ 3.0ng/ml

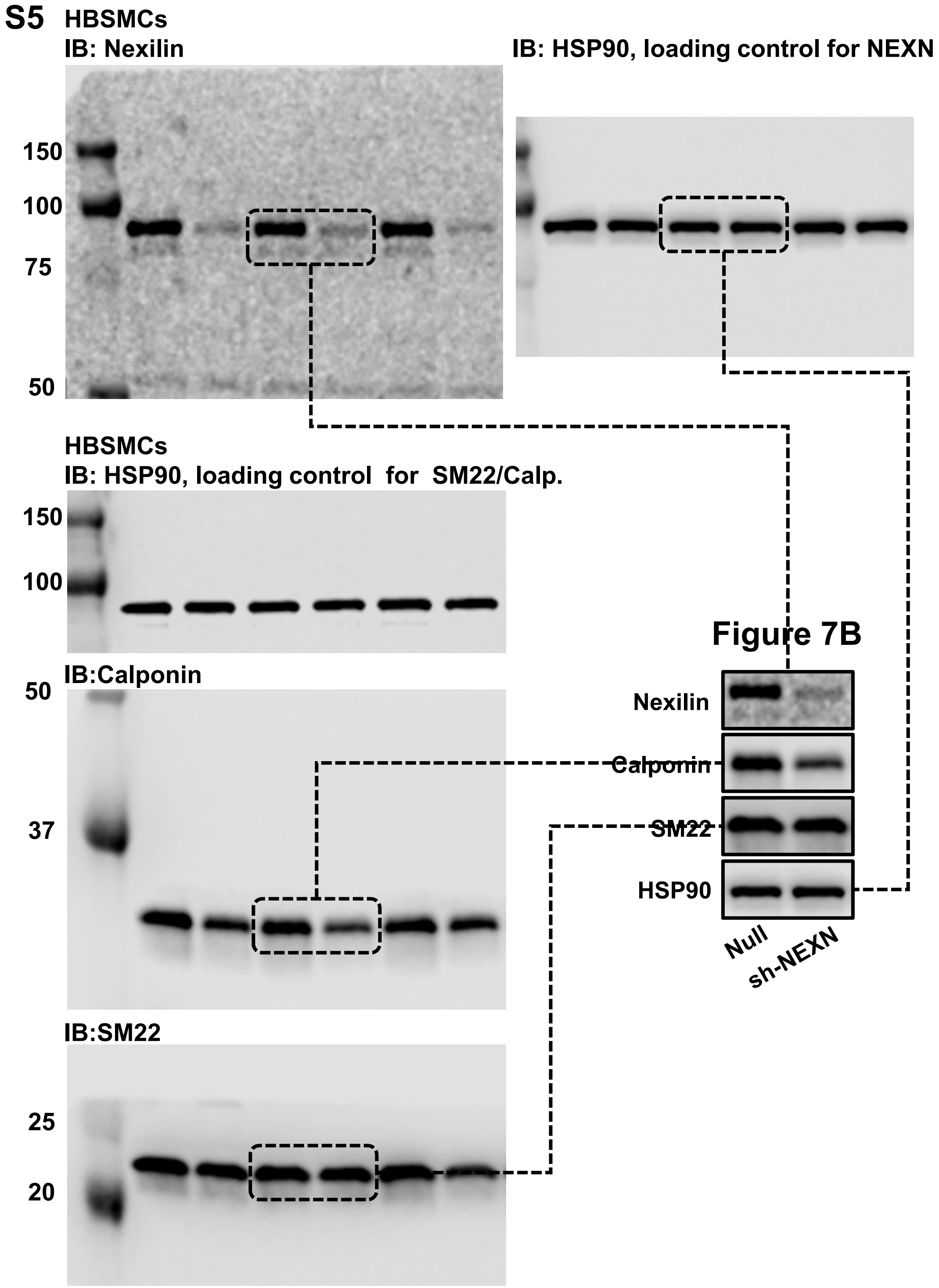
S5
HBSMCs
IB: Nexilin
IB: HSP90, loading control for NEXN
150
100
75
50
HBSMCs
IB: HSP90, loading control for SM22/Calp.
150
100
IB:Calponin
50
37
IB:SM22
25
20
Figure 7B
Nexilin
Calponin
SM22
HSP90
Null
sh-NEXN

S6

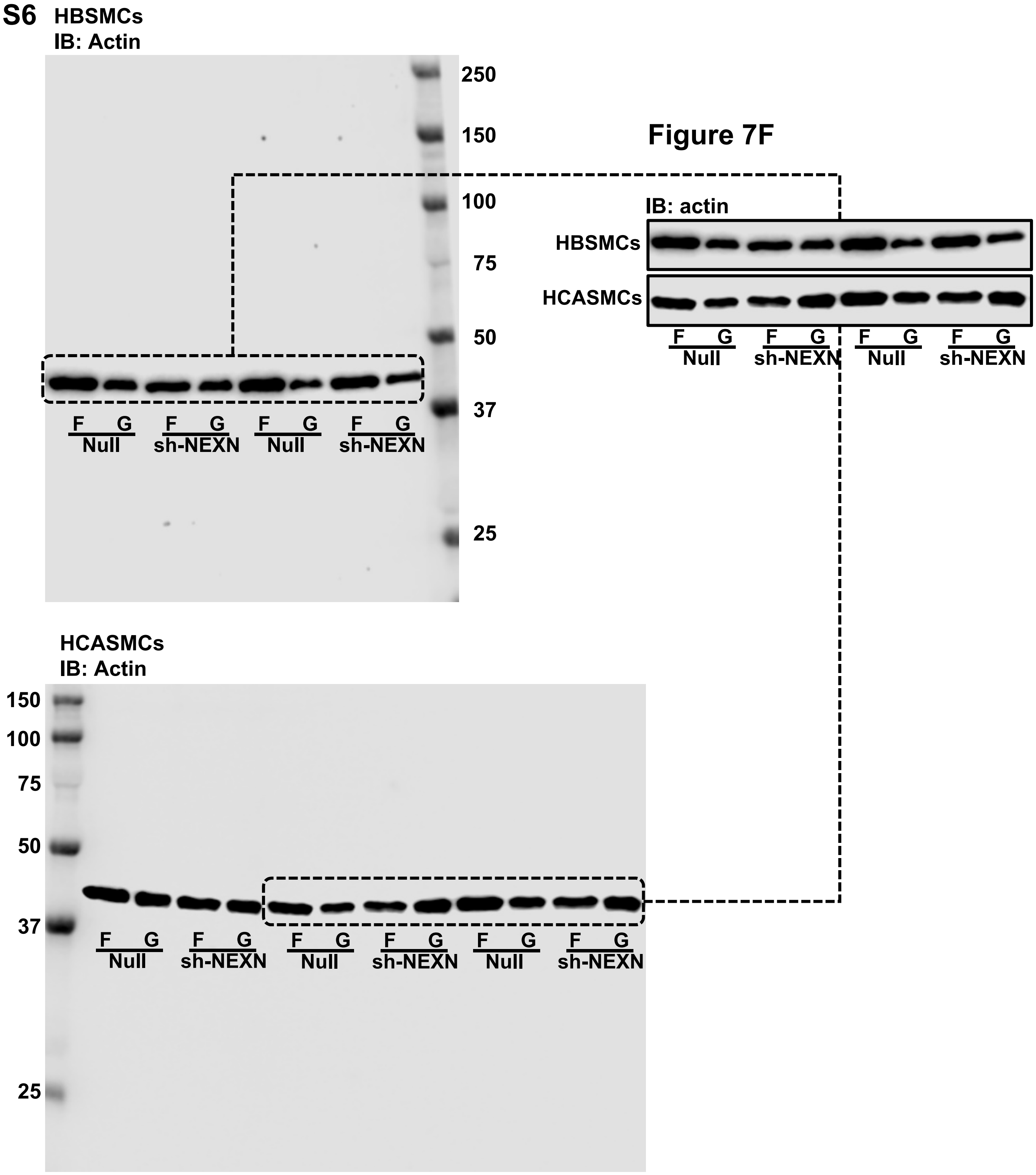

HBSMCs
IB: Actin
250
150
100
75
50
37
25
F G F G F G F G
Null sh-NEXN Null sh-NEXN
Figure 7F
IB: actin
HBSMCs
HCASMCs
F G F G F G F G
Null sh-NEXN Null sh-NEXN
HCASMCs
IB: Actin
150
100
75
50
37
25
F G F G F G F G F G F G
Null sh-NEXN Null sh-NEXN Null sh-NEXN

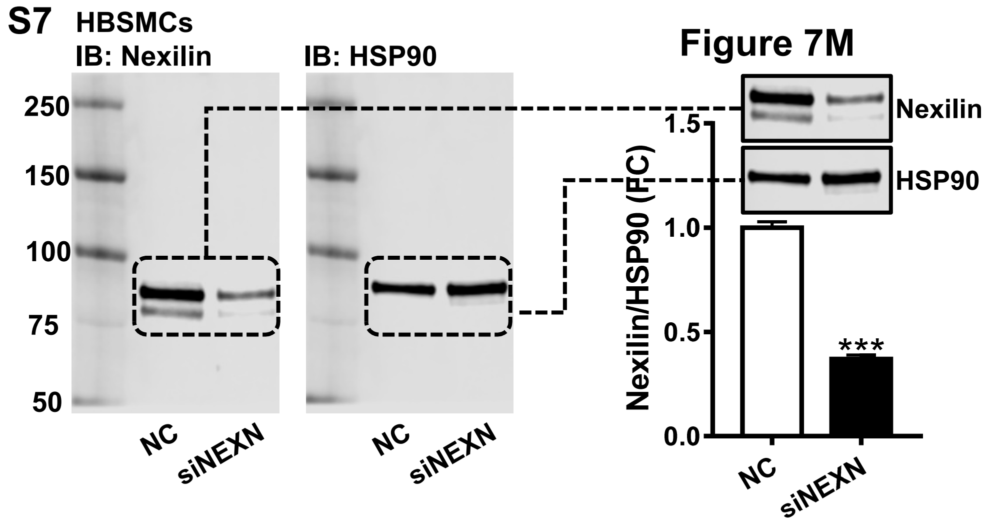
S7
HBSMCs
IB: Nexilin
IB: HSP90
250
150
100
75
50
NC
siNEXN
NC
siNEXN
Figure 7M
Nexilin
HSP90
Nexilin/HSP90 (FC)
1.5
1.0
0.5
0.0
***
NC
siNEXN
